# Differences in the setting of acetabular component alignment guides between the supine and lateral positions for total hip arthroplasty

**DOI:** 10.1038/s41598-021-01420-1

**Published:** 2021-11-09

**Authors:** Yukihide Minoda, Ryo Sugama, Yoichi Ohta, Susumu Takemura, Nobuo Yamamoto, Tamotsu Nakatsuchi, Hiroaki Nakamura

**Affiliations:** 1 Department of Orthopaedic Surgery, Graduate School of Medicine, Osaka City University, 1-4-3 Asahi-machi, Abeno-ku, Osaka, 545-8585 Japan; 2 Tsuji-Geka Rehabilitation Hospital, 3-24 Ikutamamae-machi, Tennouji-ku, Osaka, 543-0072 Japan

**Keywords:** Diseases, Medical research

## Abstract

The acetabular component orientation in total hip arthroplasty is of critical importance to clinical results. Although navigation systems and surgical robots have been introduced, most surgeons still use acetabular component alignment guides. This study aimed to compare the accuracy between modern acetabular component alignment guides for the lateral position and those for the supine position. Thirteen alignment guides for the lateral position and 10 for the supine position were investigated. All the lateral position alignment guides indicated cup alignment in operative definition, and the supine position alignment guides indicated cup alignment in radiographic definition. For lateral position alignment guides, the anteversion actually indicated by the alignment guide itself was smaller than that indicated by the manufacturer by a mean of 6° (maximum, 9°), and the inclination actually indicated by alignment guides themselves was larger than that by the manufacturer (*p* < 0.01) by a mean of 2° (maximum, 4°). For supine position alignment guides, the inclination and anteversion indicated by the alignment guide itself were identical with those indicated by the manufacturer. The current study showed that the angles actually indicated and those stated by manufacturers were not identical for lateral position alignment guides.

## Introduction

The acetabular orientation in total hip arthroplasty (THA) affected dislocation, range of motion, polyethylene wear, pelvic osteolysis, and component migration^1–5^. The concept of “safe range” for accetabular orientation was first introduced by Lewinnek et al.^6^ They showed that the dislocation rate for acetabular orientation within an inclination of 40 ± 10° and anteversion of 15 ± 10° was lower than that outside these so-called “safe ranges.” From the point of view of dislocation, they suggested that the acetabular component should be implanted in these safe ranges^6^ in radiographic definitions^7^. Although orthopedic surgery with computer assistance, such as a navigation system, robot, and patient-specific instrument, has been introduced, acetabular component alignment guides are still mainstreamed in clinical situations because of its cost and operation time. However, mechanical alignment guides previously resulted in large errors in acetabular component orientation^6,8–11^. The pelvis moves during the fixation of the acetabular component^12,13^. The mechanical alignment guides cannot correspond to the movement of the pelvis during surgery, which causes the large error in acetabular component orientation^8,12,13^.

In addition to pelvic movement, the accuracy of acetabular component alignment guides directly influences the postoperative acetabular component orientation. Therefore, it is very important to check whether or not the acetabular component alignment guide indicates the correct angle. There are two types of acetabular component alignment guide. One is for the lateral position and the other for the supine position. Previous reports showed that the angles indicated by the manufacturer were not equal to those actually indicated by alignment guides for the lateral position and that alignment guides for the lateral position itself could be one of the factors of error in acetabular component orientation^14^. However, these alignment guides for the lateral position are obsolete and are no longer used in these days. Moreover, there has been no report to date on acetabular component alignment guides for the supine position. We hypothesized that the angles indicated by the manufacturer were not equal to the angles actually indicated not only for modern alignment guides for the lateral position but also those for the supine position. This study aimed to conduct a basic investigation on the setting of modern acetabular component alignment guides for the lateral and supine positions.

## Materials and methods

In total, 23 types of modern acetabular alignment guides from 10 manufacturers were examined. The descriptions in the manufacturers’ surgical/instrumentation booklets were assessed. Of the 23 guides, 13 were designed for the lateral position and 10 for the supine position.

**Figure 1:**
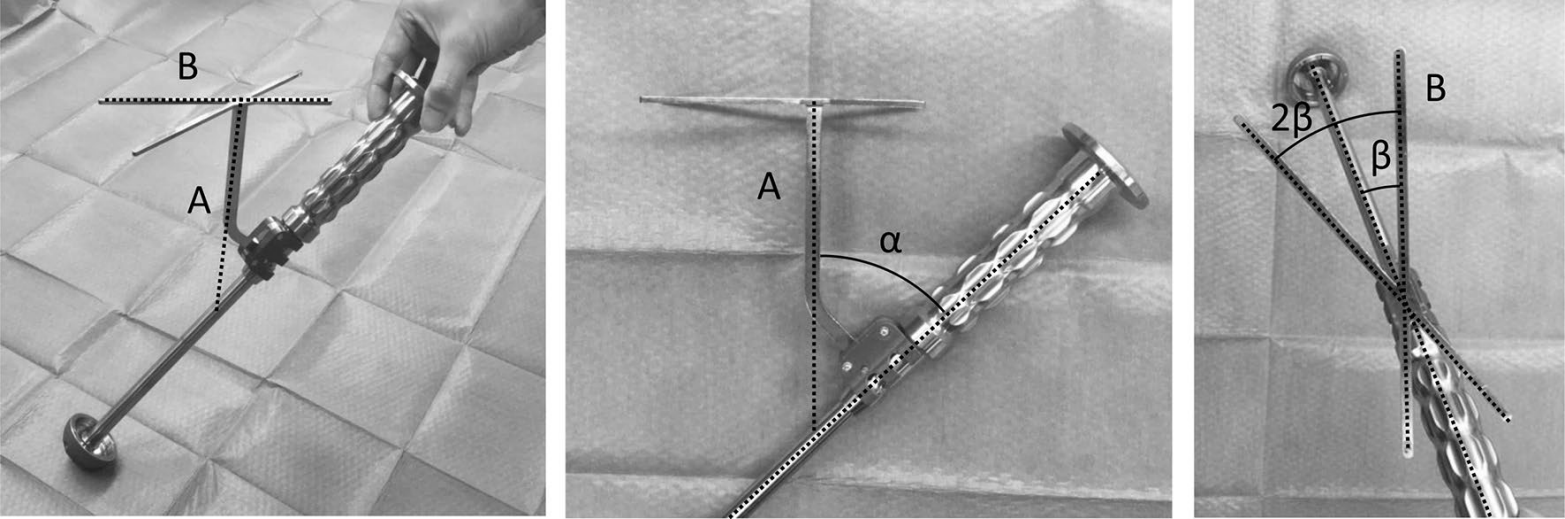
Photographs of a lateral position alignment guide. Manufacturers' surgical/instrumentation booklets described that this guide was designed for the lateral position and that line A should be vertical to the floor and line B, parallel to the long axis of the patient. The angle between the shaft of the guide and that of the X-shaped indicator was defined as α. The half angle between the X-shaped indicator bars was defined as β.

**Figure 2:**
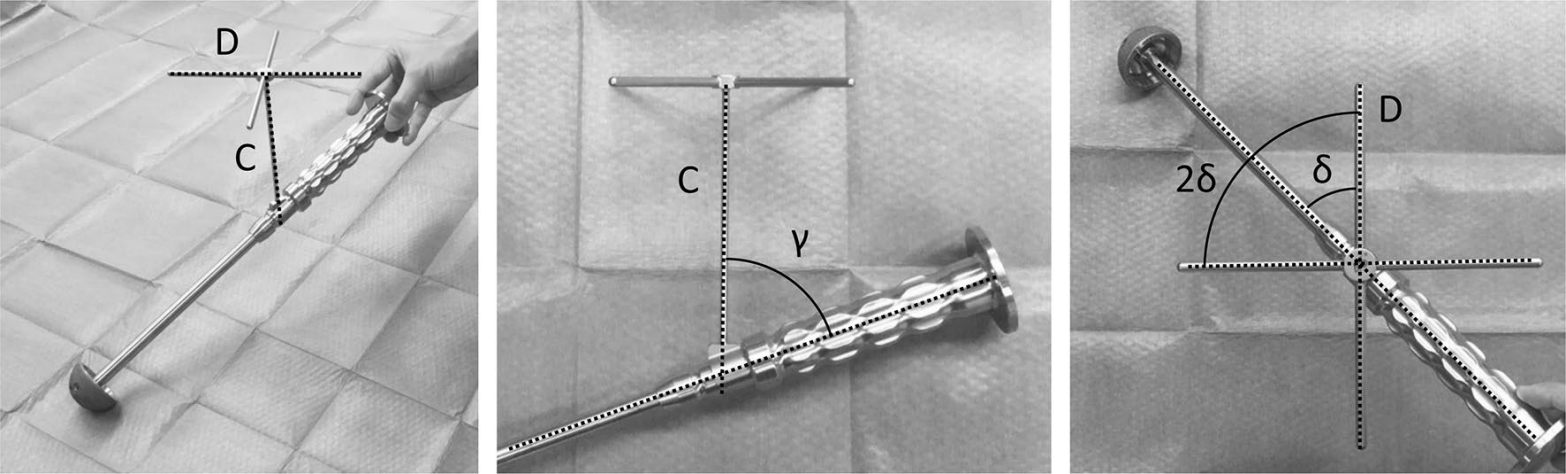
Photographs of a supine position alignment guide. Manufacturers' surgical/instrumentation booklets described that this guide was designed for the supine position and that line C should be vertical to the floor and line D, parallel to the long axis of the patient. The angle between the shaft of the guide and that of the X-shaped indicator was defined as γ. The half angle between the X-shaped indicator bars was defined as δ.

For lateral alignment guides, the angle between the shaft of the guide and the shaft of the X-shaped indicator was defined as α (Fig. 1). The half angle between the X-shaped indicator bars was defined as β (Fig. 1). For the supine alignment guides, the angle between the shaft of the guide and the shaft of the X-shaped indicator was defined as γ (Fig. 2). The half angle between the X-shaped indicator bars was defined as δ (Fig. 2). The angles between the shaft and indicator bar of each alignment guide (α, β, γ, and δ) were directly measured using a transparent protractor (Protractor for radiographic measurement of hip joint; Tosuku Inc., Saitama, Japan) according to a previous report^14^. For α and γ, a transparent protractor comprising the shaft of the cup holder and the shaft of the alignment guide was put on the plane. For β and δ, a transparent protractor comprising the X-shaped indicator was put on the plane.

The angles, whose alignment guides for the lateral and supine positions were actually indicated in radiographic definitions, were then calculated from these measured angles using modified Murray’s formulas as follows^7^:

For the lateral position alignment guide:

- Ope Inc = 90 – α
- Ope Av = β
- Rad Inc = arctan (tan [Ope Inc]/cos [Ope Av])
- Rad Av = arcsin (sin [Ope Av] × cos [Ope Inc])

For the supine position alignment guide

- Rad Inc = δ
- Rad Av = 90 − γ
- (Rad = radiographic, Ope = operative, Inc = inclination, and Av = anteversion)

The angles of inclination and anteversion indicated in the manufacturers’ surgical/instrumentation booklets were also examined. The differences between the actual alignment guide angles and those stated by the manufacturer were defined as the error of alignment guides.

The angles between the shaft and indicator bar of each alignment guide were measured by an experienced orthopedic surgeon. To evaluate the intraobserver error associated with the measuring method, the author measured twice. To evaluate the interobserver error associated with the measuring method, another experienced orthopedic surgeon measured the angles of all of the alignment guides using the same method. Interobserver and intraobserver reproducibility was assessed using the Bland–Altman method^15^ for each comparison. We defined the 95% confidence limits (CLs) (i.e., mean + /− 2 standard deviations [SDs]). Intraobserver analysis indicated a mean difference of − 0.01% (95% CLs: − 0.09%, 0.12%) in inclination and 0.27% (95% CLs: − 1.82%, 2.36%) in anteversion. Interobserver analysis indicated a mean difference of − 0.06% (95% CLs: − 0.43%, 0.55%) in inclination and 0.07% (95% CLs: − 0.47%, 0.60%) in anteversion. The Bland–Altman method^15^ showed that the intraobserver and interobserver reliabilities of inclination and anteversion were high.

## Results

For the lateral position alignment guides, the angles indicated by the manufacturer were 44 ± 2° (mean ± SD) in inclination and 21 ± 4° in anteversion (Table 1). However, the actual angles indicated by the lateral position alignment guides were 46 ± 3° in inclination and 15 ± 3° in anteversion. The inclination and anteversion actually indicated by the alignment guide itself were larger and smaller, respectively, than those indicated by the manufacturer. The errors of the lateral position alignment guides were 2 ± 1° for inclination and 6 ± 2° for anteversion. The definition of all the lateral position alignment guides was operative. However, the definition was not indicated in the manufacturers’ surgical/instrumentation booklets.

**Table 1:** Inclination and anteversion of lateral position alignment guides.

Manufacturer	Product name	Definition	Was definition indicated in manufacturers’ surgical/instrumentation booklets?	Inclination			Anteversion		
				Angle stated by manufacturer	Angles actually indicated by alignment guide	Error of alignment guide	Angle stated by manufacturer	Angles actually indicated by alignment guide	Error of alignment guide
AceClap	Plasmafit	Operative	No	40	41	1	15	11	− 4
Adler	Fixa Ti-Por	Operative	No	45	47	2	20	14	− 6
DePuy	Pinnacle								
	MACS offset impactor	Operative	No	45	49	4	30	21	− 9
	Pinnacle straight impactor	Operative	No	45	49	4	30	21	− 9
	Offset cup impactor	Operative	No	40	42	2	20	15	− 5
Kyocear	SQRUM	Operative	No	40	42	2	20	15	− 5
Medacta	Mpact	Operative	No	40	42	2	20	15	− 5
Microport	Dynasty	Operative	No	45	47	2	20	14	− 6
Smith&Nephwe	R3								
	Offset shell impactor	Operative	No	45	47	2	20	14	− 6
	Standard offset	Operative	No	45	47	2	20	14	− 6
Stryker	Trident	Operative	No	45	47	2	20	14	− 6
ZimmerBiomet	Continuum	Operative	No	45	47	2	20	14	− 6
	G7	Operative	No	40	42	2	20	15	− 5
Mean ± SD				44 ± 2	46 ± 3	2 ± 1	21 ± 4	15 ± 3	− 6 ± 2

*SD* standard deviation.

For the supine position alignment guides, the angles indicated by the manufacturer were 44 ± 2° in inclination and 18 ± 3° in anteversion (Table 2). The angles actually indicated by the supine position alignment guide itself were 44 ± 2° in inclination and 18 ± 3° in anteversion. The angles actually indicated and those stated by the manufacturers were the same. There were no differences between the angles indicated by the alignment guide itself and those indicated by the manufacturer. The error of the supine position alignment guides was 0° for inclination and anteversion. The definition of all the supine position alignment guides was radiographic. However, the definition was not indicated in the manufacturers’ surgical/instrumentation booklets.

**Table 2:** Inclination and anteversion of supine position alignment guides.

Manufacturer	Product name	Definition	Was definition indicated in Manufacturers’ surgical/instrumentation booklets?	Inclination			Anteversion		
				Angle stated by manufacturer	Angles actually indicated by alignment guide	Error of alignment guide	Angle stated by manufacturer	Angles actually indicated by alignment guide	Error of alignment guide
AceClap	Straight	Radiographic	No	40	40	0	15	15	0
Adler	Fixa Ti-Por	Radiographic	No	45	45	0	20	20	0
DePuy	Pinnacle								
	Offset cup impactor	Radiographic	No	40	40	0	15	15	0
Kyocear	SQRUM	Radiographic	No	40	40	0	20	20	0
Medacta	Mpact	Radiographic	No	40	40	0	20	20	0
Smith&Nephwe	R3								
	Offset shell impactor	Radiographic	No	40	40	0	15	15	0
	Standard offset	Radiographic	No	40	40	0	15	15	0
Stryker	Trident	Radiographic	No	45	45	0	20	20	0
ZimmerBiomet	Continuum	Radiographic	No	45	45	0	20	20	0
	G7	Radiographic	No	40	40	0	20	20	0
Mean				42 ± 3	42 ± 3	0 ± 0	18 ± 3	18 ± 3	0 ± 0

*SD* standard deviation.

## Discussion

The most important finding in the present study was that the angles indicated by the manufacturer were equal to those actually indicated by modern alignment guides for the supine position but were not equal to those actually indicated by modern alignment guides for the lateral position. The lateral alignment guides were designed with the operative definition. The supine position alignment guides were designed with the radiographic definition.

For modern acetabular component alignment guides for the lateral position, the angles indicated by the manufacturer were not equal to those actually indicated by the alignment guide itself. The modern alignment guides for the lateral position, which we use in clinical settings, inherently mislead anteversion to a decrease by a mean of 6° (maximum: 9°) and inclination to an increase by a mean of 2° (maximum: 4°) because the lateral position alignment guides were designed with the operative definition. The present study suggested that the use of lateral position alignment guides would not eliminate the errors in acetabular component orientation, even if intraoperative pelvic motion was perfectly controlled. The modern lateral position alignment guides themselves were the risk factor for errors in acetabular component orientation, especially in anteversion. To eliminate this type of error, the lateral position alignment guides should be redesigned not with the operative definition but with the radiographic definition. The supine position alignment guides were designed with the radiographic definition. Therefore, the alignment guides for the supine position themselves cannot be factors in the incidence of errors in acetabular component orientation. From the point of view of the setting of the modern alignment guides, the alignment guides for the supine position are advantageous.

A previous report^14^ investigated the lateral position alignment guides of old models that are currently rarely used. Surprisingly, the differences between the angles indicated by the manufacturer and those actually indicated by the alignment guide itself were the same for old models in previous reports and modern models in the current study. This study also showed that the setting of alignment guides for the lateral position has not improved at all in the last 10 years. Surgeons and manufacturers should be aware of this situation.

Yoshitani et al. investigated the acetabular component orientation in 181 THAs using the lateral position^16^. They used lateral position alignment guides, which were set at 40° for inclination and 20° for anteversion, in all of their cases and set the acetabular component according to the alignment guide. However, the average of the postoperative acetabular component orientation was 41° for inclination and 14° for anteversion. The lateral position alignment guide that Yoshitani J. et al. used was designed at 40° for inclination and 20° for anteversion in the operative definition; thus, that alignment guide theoretically set the acetabular component at 42° for inclination and 15° for anteversion in the radiographic definition^16^. Therefore, the inclination was increased and anteversion was decreased. These reports suggested that the lateral position alignment guides should be used with caution.

The clinically significant finding of this basic study was that the modern alignment guides for the lateral position inherently mislead anteversion to a decrease by a mean of 6° (maximum: 9°) and inclination to an increase by a mean of 2° (maximum: 4°). This type of error could theoretically be resolved by “reducing” inclination and “adding” anteversion during the operation with consideration of the error of the alignment guide. However, such adjustments are not required for supine position alignment guides. Before the operation, surgeons should be aware of the definition of alignment guides and the “true angle” that the alignment guides indicate.

This study had limitations. First, this was not a clinical study. Cup alignment is influenced not only by alignment guides but also by other factors such as patient characteristics, surgeons’ skill, surgical approach, type of pelvic positioner, and preoperative planning. This study could not evaluate such factors. However, this study showed that the design of the modern alignment guides can be one of the risk factors for cup alignment errors in the lateral position and not in the supine position. Second, the clinical impact of a mean 6° error in anteversion and a mean 2° error in inclination for the modern lateral position alignment guides was not evaluated. To assess this issue, a clinical comparative study should be conducted using lateral position alignment guides with the operative definition and the radiographic definition. However, it is impossible to perform such studies because there are currently no lateral position alignment guides with the radiographic definition.

The current study showed that the angles actually indicated and those stated by manufacturers were not identical in modern alignment guides for the lateral position. Such a setting of modern alignment guides could result in one of the risk factors in the error of acetabular component orientation. The definition of acetabular alignment guides should be checked before their use.
